# Role of apolipoprotein C1 in lipoprotein metabolism, atherosclerosis and diabetes: a systematic review

**DOI:** 10.1186/s12933-022-01703-5

**Published:** 2022-12-05

**Authors:** Alexia Rouland, David Masson, Laurent Lagrost, Bruno Vergès, Thomas Gautier, Benjamin Bouillet

**Affiliations:** 1 Endocrinology and Diabetology Unit, University Hospital, Dijon, France; 2 INSERM/University of Bourgogne Franche-Comté, LNC UMR1231, Dijon, France; 3 LipSTIC LabEx, UFR Sciences de Santé, Dijon, France; 4 Service Endocrinologie, Diabétologie et Maladies Métaboliques, Hôpital François Mitterrand, CHU Dijon, BP 77908, 21079 Dijon, France

**Keywords:** Apolipoprotein C1, Diabetes, Atherosclerosis, Lipoprotein metabolism, Triglyceride-rich lipoproteins, High density lipoproteins, Cholesteryl ester transfer protein

## Abstract

Apolipoprotein C1 (apoC1) is a small size apolipoprotein whose exact role is not totally clarified but which seems to modulate significantly the metabolism of lipoproteins. ApoC1 is involved in the metabolism of triglyceride-rich lipoproteins by inhibiting the binding of very low density lipoproteins (VLDL) to VLDL-receptor (VLDL-R), to low density lipoprotein receptor (LDL-R) and to LDL receptor related protein (LRP), by reducing the activity of lipoprotein lipase (LPL) and by stimulating VLDL production, all these effects leading to increase plasma triglycerides. ApoC1 takes also part in the metabolism of high density lipoproteins (HDL) by inhibiting Cholesterol Ester Transfer Protein (CETP). The functionality of apoC1 on CETP activity is impaired in diabetes that might account, at least in part, for the increased plasma CETP activity observed in patients with diabetes. Its different effects on lipoprotein metabolism with a possible role in the modulation of inflammation makes the net impact of apoC1 on cardiometabolic risk difficult to figure out and apoC1 might be considered as pro-atherogenic or anti-atherogenic depending on the overall metabolic context. Making the link between total plasma apoC1 levels and the risk of cardio-metabolic diseases is difficult due to the high exchangeability of this small protein whose biological effects might depend essentially on its association with VLDL or HDL. The role of apoC1 in humans is not entirely elucidated and further studies are needed to determine its precise role in lipid metabolism and its possible pleiotropic effects on inflammation and vascular wall biology. In this review, we will present data on apoC1 structure and distribution among lipoproteins, on the effects of apoC1 on VLDL metabolism and HDL metabolism and we will discuss the possible links between apoC1, atherosclerosis and diabetes.

## Introduction

ApoC1 is the smallest plasma apolipoprotein, composed of 57 amino-acids only. Its very simple structure, together with the analysis of sequence similarities, suggest that apoC1 reflects most closely the ancestor gene from which derived many other apolipoproteins (apo) such as apoC2, apoC3 and apoE [1]. While the involvement of the three latter in the regulation of plasma triglyceride hydrolysis and lipoprotein remnant uptake has been extensively studied and clearly established over years, apoC1 has long appeared as the “poor relative” of the family. Although its similarities with the ApoE/C2/C4 group initially oriented investigations toward a role in triglyceride-rich lipoprotein (TRL) metabolism [2], apoC1 appears to have a wider impact on different lipoprotein classes, as reflected by its association with HDL (80%) and to a lesser extent with VLDL (20%) in normolipidemic plasma [3, 4]. Accordingly, a substantial amount of studies demonstrated that, beyond its impact on VLDL and chylomicron metabolism, apoC1 plays a key role in the regulation of HDL remodelling [5–11]. This wide range of effects together with a possible role in the modulation of inflammation makes the net impact of apoC1 on cardiometabolic risk difficult to figure out and apoC1 might be considered as pro-atherogenic or anti-atherogenic depending on the overall metabolic context. Investigations of the functions of apoC1 in animal models can somehow be complex, since most rodent models substantially differ from humans concerning several features of lipoprotein metabolism, especially the absence of active CETP, i.e. one of the main target of apoC1 in humans [12–14]. Genetic studies in humans are also hampered by the fact that apoC1 gene polymorphisms are tightly linked to apolipoprotein E variants [15–18]. In addition, making the link between total plasma apoC1 levels and the risk of cardio-metabolic diseases is difficult due to the high exchangeability of this small protein whose biological effects might depend essentially on its association with VLDL or HDL. In this review we will present data on apoC1 structure and distribution among lipoproteins, on the effects of apoC1 on VLDL metabolism and HDL metabolism and we will discuss the possible links between apoC1, atherosclerosis and diabetes.

## ApoC1 structure and gene

**Fig. 1:**
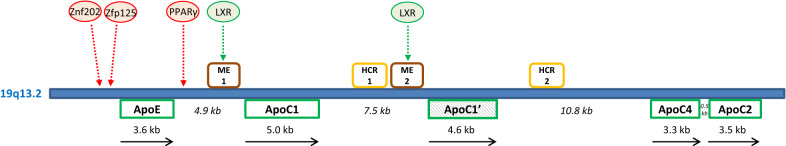
Position of apoC1 gen on chromosome 19 within a cluster encompassing the apoE, apoC1’, apoC2 and apoC4 genes. Genes are shown in green boxes, with their respective length indicated below. Black arrows indicate gene orientation. The length of noncoding regions between genes are indicated in italic. ApoC1’ pseudogene is hatched in grey. Regulatory regions are shown in brown (ME 1 and ME2) and yellow boxes (HCR1 and HCR2). ME and HCR regions promote gene expression in macrophages and hepatocyte, respectively. Transcription factors involved in the regulation of APOC1 gene are shown in ovals, with activators in green and repressors in red. *Kb* kilobase, *ME* multienhancer, *HCR* hepatic control region, *LXR* liver X receptor, *PPAR* peroxisome proliferator-activated receptor, *Znf, Zfp* zinc finger protein

**Fig. 2:**
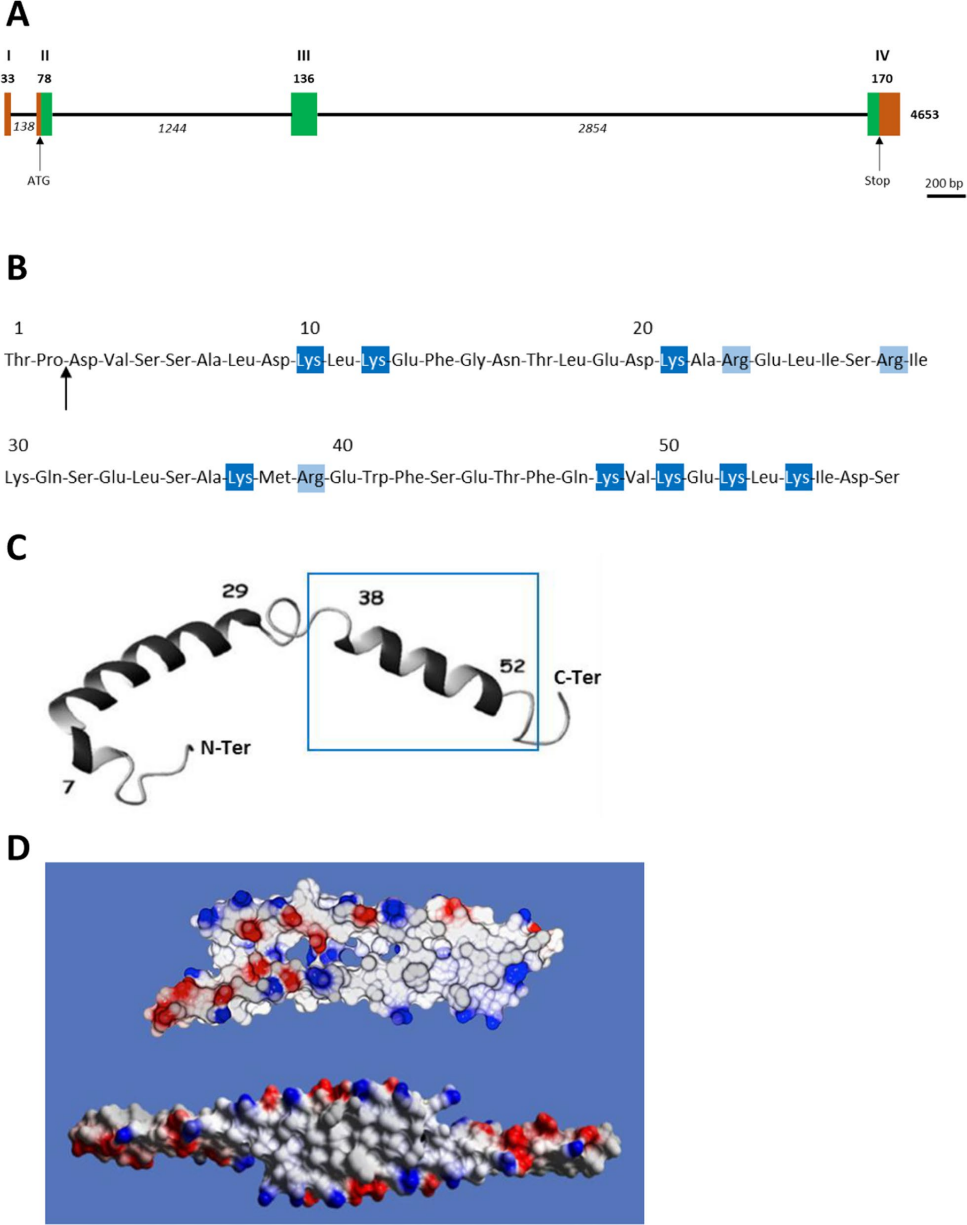
Schematic representation of apoC1. **A** Structure of the transcribed region of the apoC1 gene. Exons are shown as boxes numbered with Roman numerals. Bold numbers are exon lengths, in nucleotides. Italicized numbers are intron lengths. The total length is indicated at the 3’-end of the gene. Beginning (ATG) and end (Stop) of translation are shown with black arrows. Untranslated and translated exonic sequences are shown in brown and green, respectively. **B** Primary structure of apoC1. Basic aminoacid residues are highlighted in dark blue for lysine and light blue for arginine. A lysine-rich cluster spans from residues 48 to 54. The vertical arrow represents the described possible site of truncation of apoC1 [35]. **C** Conformation of apoC1 with 2 α-helices according to [36] (permission validated by editor). The N-terminal helix spans from residues 7 to 29, the C-terminal helix from residue 38 to 52. The two helices are separated by an unstructured, hinge region. The conformation according to 2 or one α-helix might depend on the hydrophobicity of the environment. The blue frame delineates the region associated with the CETP-inhibitory effect. **D** Conformation of apoC1 with one α-helix with two possible dimeric associations according to [37] (permission validated by editor). Positively charged residues are shown in blue; negatively charged residues are shown in red. Hydrophobic clusters resulting from dimerization are visible as white/grey patches

The human apoC1 gene is located on chromosome 19 (locus 19q13.2) [1, 19] within a 45-kilobase cluster encompassing also the apoE, apoC1’, apoC2 and apoC4 genes (Fig. 1). It is located 4.3 kb [20–22] or 5.3 kb [19] downstream from the apoE gene, in the same transcriptional orientation. A copy of the apoC1 gene, apoC1’ is located 7.5 kb downstream from the apoC1 gene (Fig. 1). The apoC1 gene is 4653 base pairs long while the apoC1’ gene is 4387 base pairs long. Both genes contain 4 exons and 3 introns (Fig. 2). No mRNA product of the apoC1’ gene can be detected in any tissue, suggesting that it is a pseudogene. The similar structure and the proximity of apoC1 with apoE, C1’, C2 and C4 genes suggest that they are derived from a common ancestor gene, with a primordial apolipoprotein gene that was most likely very similar to current apoC1 [1].

The regulation apoC1 gene expression is under the control of regulatory elements located throughout the ApoE/C1/C1’/C2/C4 cluster. These include, among others, two copies of hepatic control regions (HCR-1 and HCR-2) and two copies of multienhancers (ME-1 and ME-2) (Fig. 1). While HCR sequences are required for the expression of apoC1 gene in hepatocytes [23], ME-1 and ME-2 play a significant role in stimulating its expression in macrophages through the binding of the Liver X Receptor (LXR) via LXR response elements [24]. On the opposite, a Peroxisome Proliferator-activated Receptor (PPAR) gamma response element located the intergenic region between apoE and apoC1 was shown to be involved in a negative effect of glitazones on apoC1 gene expression in hepatocytes [25, 26]. Finally, apoC1 gene expression was also shown to be downregulated by zinc-finger proteins Znf202 and Zfp125 which play a role in the regulation of genes involved in HDL metabolism and hepatic cholesterol /bile acid elimination on the one hand, and hepatic steatosis and VLDL secretion on the other hand [27, 28]. Importantly, all cis- and trans- regulatory elements mostly exert their effects not only on the apoC1 gene alone but on the whole ApoE/C1/C1’/C2/C4 gene cluster [23, 24, 26–28]. Overall, although apoC1 gene is expressed in many tissues such as lung, skin, testes, spleen, and adipose tissue, the main sites for its expression are the liver and differentiated macrophages [19, 24, 29, 30]. In addition to regulation of apoC1 gene expression at the transcriptional level, the secretion of apoC1 protein can also be modulated post-transcriptionally, with a stimulatory effect in the context of increased cholesterol pool, while cellular triglyceride content had no effect [31].

ApoC1 is synthesized with a 26-residue signal peptide, which is cleaved cotranslationally in the rough endoplasmic reticulum [32]. The mature protein encompasses 57 amino acids for a total molecular weight of 6613 Da, which makes apoC1 the smallest of all circulating apolipoproteins [33, 34] (Fig. 2). In addition, due to its high relative content in basic amino acid residues (7 lysine, 3 arginine), apoC1 is the apolipoprotein with the highest isoelectric point (pH(I) = 8.3) [33, 34]. On the opposite, apoC1 contains no histidine, tyrosine, or cysteine residues (for complete primary structure, see Fig. 2). A truncated form of apoC1, devoid of the N-terminal threonine and proline residues could also be detected in plasma and might result from the action of plasma dipeptidyl peptidase IV [35]. While initial nuclear magnetic resonance (NMR) studies indicated that apoC1 is composed of two amphipathic α-helices (residues 7–29 and 38–52) [36] more recent investigations using X-ray diffraction on apoC1 crystals suggest that the whole molecule can form a single α-helix [37] (Fig. 2). The conformation according to 2 or one α-helix might depend on the hydrophobicity of the environment [36, 37]. The amphipathic properties are important for the affinity of apoC1 for different lipoprotein classes [36, 38, 39] and might also influence its exchangeability between them [40]. Interestingly, apoC1 proteins have the ability to form dimers, or to fold their two α-helices upon one another [37, 41]. Mature apoC1 is devoid of glycosylation and, due to the absence of cysteine residues, of disulphide bonds.

## Plasma levels and distribution of apoC1 between lipoproteins

The serum concentration of apoC1 is about 60 mg/L in normolipidemic subjects [3, 13, 42–44]. ApoC1 is mainly measured by enzyme-linked immunosorbent assay (ELISA) [13, 45, 46] but the use of mass spectrometry is becoming more and more widespread [47]. ApoC1 is mainly associated with HDL and VLDL in humans [4, 48, 49]. Methodological reports raised the possibility that small, exchangeable apolipoproteins, such as apoC1 [2, 3], might undergo shedding or redistribution between lipoprotein classes especially during extensive ultracentrifugation procedures [50]. Pioneering studies reported that up to 14% of apolipoproteins C are shed from VLDL after 2 ultracentrifugation runs [51], and the recovery of labeled apoC1 in the lipoprotein-free fraction after ultracentrifugation (less than 10% of total pool) was reported in kinetic studies [4].

In normolipidemic subjects, the majority of apoC1 is associated with HDL (80% to 95%) [3, 4]. Additionally, substantial amounts of apoC1 could also be found in chylomicrons in the post-prandial phase [52, 53].

In hyperlipidemic individuals, several groups reported increased plasma levels of apoC1, especially in the context of hypertriglyceridemia or mixed hyperlipidemia, with values reaching 200 mg/L in some studies [3, 43, 45]. However, this phenotype was not always observed [13]. Hypertriglyceridemia or mixed hyperlipidemia, were also associated with alterations in the distribution of apoC1 between lipoproteins, i.e. an enrichment in VLDL at the expense of HDL. Indeed, 49% of apoC1 is associated with VLDL in subjects with hypertriglyceridemia [3] and 43% in subjects with combined hyperlipidemia (high level of triglycerides and LDL cholesterol), whereas the relative amounts of apoC1 in HDL decreased (respectively 51% and 57%) [3]. This relative enrichment of VLDL during hypertriglyceridemia has been proposed to result from 1) increased hepatic apoC1 production coupled with enhanced VLDL secretion and/or 2) redistribution from HDL to VLDL in the circulation [3] since apoC1 has been demonstrated to be a highly exchangeable protein [4, 40].

## Role of apoC1 in the VLDL and chylomicron metabolism

Pioneering in vitro and ex vivo studies demonstrated that apoC1 impairs the clearance of triglyceride-rich lipoproteins (TRLs). It was shown, in vitro, in isolated-perfused rat liver models, that adding human apoC1 to chylomicrons [54] or triglycerides (TG) emulsions [55] led to the inhibition of their hepatic uptake. It was then shown that apoC1 inhibited the apoE-dependent binding of β-VLDL to LRP [56, 57]. It was suggested that this inhibiting action of ApoC1 on the binding of lipoproteins to LRP was related to the displacement of apoE from the lipoproteins since apoC1 peptides were able to displace significant quantities of apoE from β-VLDL and to inhibit the binding of β-VLDL to LRP [58]. In addition, apoC1 could impair the apoE-mediated binding of VLDL to the LDL-R, either by masking the apoE, or by changing the conformation of apoE [59, 60]. Altogether, these studies led to the conclusion that apoC1 inhibited hepatic uptake of TRLs via the impaired binding of these lipoproteins either to LDL-R or to LRP by impairing their interaction via apoE.

The inhibitory effect of apoC1 on TRL clearance could be confirmed in vivo in different transgenic mouse models overexpressing human apoC1*.* All of these transgenic mice displayed an increase in their plasma triglyceride levels and to a lesser extent an increase in their plasma levels of total cholesterol [61–63]. One study showed that the increase in plasma triglyceride levels occurred in a dose-dependent manner according to the expression of the apoC1 transgene [64]. This hypertriglyceridemic phenotype was related to a considerable increase in VLDL levels [63, 65]. It was then shown in VLDL turnover studies that the clearance of VLDL from the circulation was less efficient in apoC1 transgenic mice as compared with control mice. Hepatic VLDL production and extrahepatic lipolysis was not different between mice overexpressing human apoC1 and control mice [65]. These data suggest that the hypertriglyceridemia in apoC1 transgenic mice results primarily from impaired hepatic VLDL particle uptake, in line with the in vitro observations. However, the inhibition of VLDL uptake in mice overexpressing human apoC1 was also observed in the context of LDL-R deficiency, suggesting that apoC1 inhibition of VLDL uptake involves another pathway than LDL-R, probably LRP [65]. In addition, it was shown that the overexpression of the VLDL-R in apoC1 transgenic mice had no effect on the hyperlipidemia [66]. This suggests that apoC1 also inhibits the binding of lipoproteins to the VLDL-R, which was also supported by in vitro studies [66].

Inhibition of the apoE-mediated hepatic uptake of VLDL is not the sole mechanism accounting for the hypertriglyceridemic effect of apoC1 overexpression since apoC1 transgenic mice also develop hypertriglyceridemia in an apoE-deficient context [8, 67]. Reduced lipoprotein lipase (LPL) activity might account for this effect since it was shown in vitro that apoC1 could inhibit LPL [68]. This inhibitory effect of apoC1 on LPL activity was confirmed in vivo in transgenic mice models [67] and was shown to be independent from apoC3 (physiological inhibitor of LPL) and VLDL-R (known modulator of LPL) [69]. The role of apoC1 in inhibiting LPL activity was further confirmed in humans in 28 subjects with kidney failure and on haemodialysis [70]. In this population, the dialysis procedure led to apoC1 depletion in VLDL, making them better substrates for LPL ex vivo. Accordingly, the apoC1 content of VLDL was shown to correlate negatively with LPL activity in 81 aged volunteers [71]. Finally, one study conducted in mice deficient for apoE and expressing apoC1 at physiological levels found that apoC1 increased the production rate of hepatic VLDL-TG and VLDL-apoB [64]. This observation is consistent with increased production rate of apoC1 in hypertriglyceridemic individuals [3], although the causal link between both phenomena was not investigated in this clinical study.

**Fig. 3:**
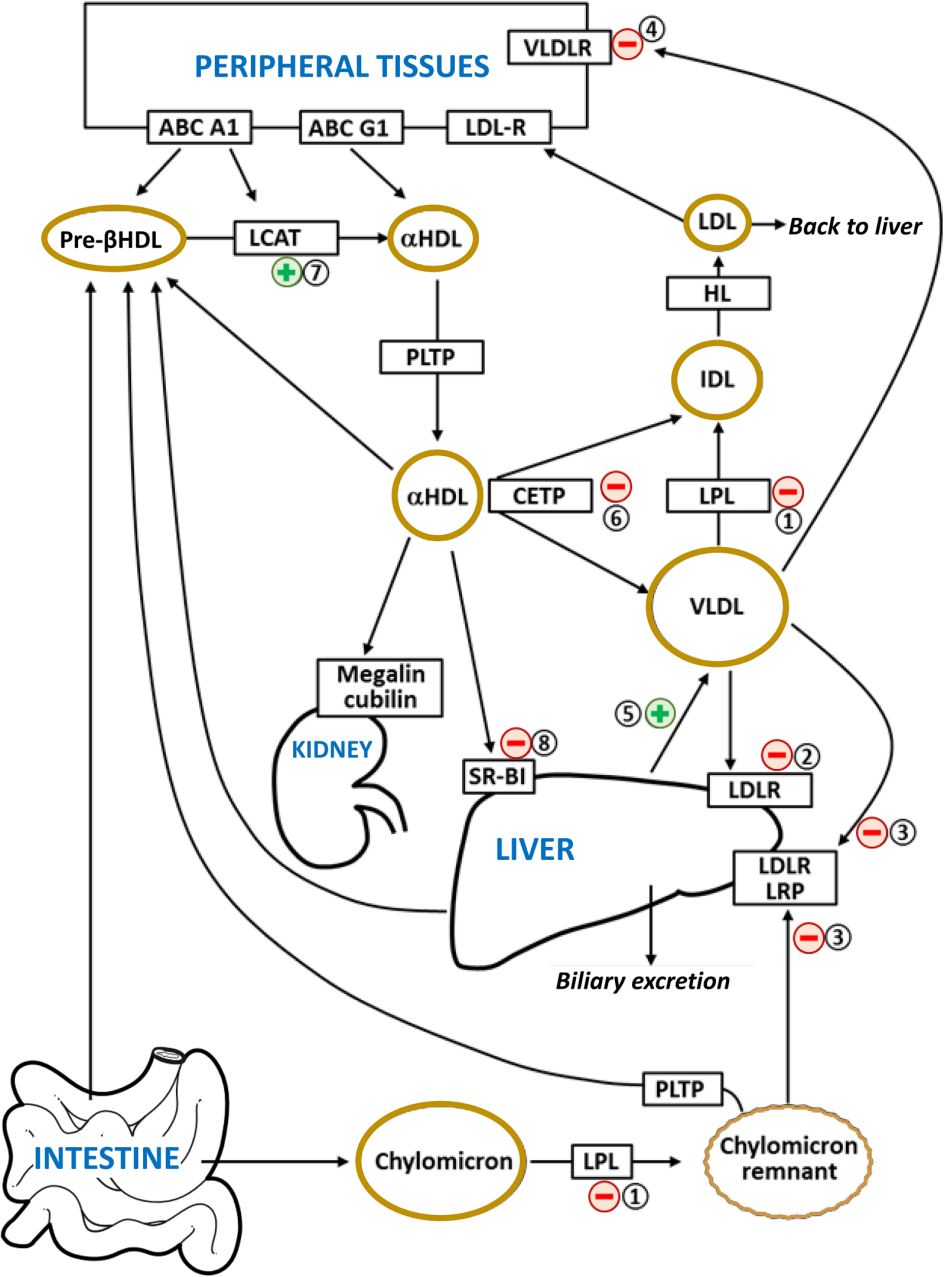
Influence of apoC1 on lipoprotein metabolism. ApoC1 acts on the metabolism of triglyceride-rich lipoproteins, leading to an increase in plasma TG levels by promoting TRL secretion by and inhibiting TG hydrolysis as well as TRL remnant clearance. ApoC1 also acts on the metabolism of HDL by favouring HDL maturation and by limiting the net loss of cholesteryl esters toward VLDL and LDL. (1) ApoC1 inhibits LPL activity. (2) ApoC1 inhibits the binding of triglyceride-rich lipoproteins to LDL-R. (3) ApoC1 inhibits the binding of triglyceride-rich lipoproteins to LRP. (4) ApoC1 inhibits the binding of triglyceride-rich lipoproteins to VLDL-R. (5) ApoC1 stimulates the hepatic production of VLDL. (6) ApoC1 is the physiological inhibitor of CETP. (7) ApoC1 stimulates LCAT activity. (8) ApoC1 inhibits SR-BI. *ABCA1* ATP-binding cassette A1, *ABCG1* ATP-binding cassette G1, *CETP* cholesteryl ester transfer protein, *HDL* high density lipoprotein, *HL* hepatic lipase, *IDL* intermediate density lipoprotein, *LCAT* lecithin-cholesterol acyltransferase, *LDL* low density lipoprotein, *LDL-R* LDL receptor, *LPL* lipoprotein lipase, *LRP* LDLR related protein, *PLTP* phospholipid transfer protein, *VLDL* very low density lipoprotein, *VLDL-R* VLDL receptor

Overall, apoC1 affects VLDL metabolism (Fig. 3) as shown by the marked increase in plasma TG levels and the lesser increase in plasma levels of total cholesterol observed in transgenic mice expressing human apoC1. Many data suggest that apoC1 could directly or indirectly inhibit LPL activity, leading to a decrease on the clearance of VLDL. Interestingly, these effects are very similar to the widely documented impact of another small apolipoprotein which is present in high amounts in TRL, apoC3, on TG levels. This might suggest that both proteins could have overlapping functions (see Table 1). In addition, this lipid phenotype could be explained by the inhibitory effect of apoC1 on hepatic receptors LDL-R and LRP, which leads to a decrease in the hepatic uptake of VLDL. However, it should be borne in mind that LDL-R and LRP- mediated uptake is far from being the main pathway for VLDL clearance in humans. In addition, these effects on hepatic receptors have been shown only in animal models expressing apoC1 at supra-physiological levels. Furthermore, one study in a knock-out mouse model on a high-fat, high-cholesterol diet showed that apoC1 deficiency also led to decreased fractional catabolic rate of VLDL [72] while a recent study using transgenic rabbits expressing human apoC1 failed to observe an increase in plasma TG levels on a western diet [49]. These latter data further demonstrate that the impact of apoC1 on TRL metabolism is more complex than that of apoC3 which was consistently shown to exert hypertriglyceridemic effects [73]. Additional kinetic studies in human populations presenting with various clinical conditions are required to get a comprehensive view of the mechanisms involved in triglyceride homeostasis which are modulated by apoC1 in our species.

**Table 1:** Comparison of molecular characteristics and biological effects of apoC1 and apoC3

	ApoC1	Refs.	ApoC3	Refs.
Gene locus—cluster	19q13.32-APOE/C1/C4/C2	[1, 19]	11q23.3-APOA1/C3/A4/A5	[161]
Introns/exons	4/3	[20]	4/3	[161]
Regulatory factors	Activators: LXR Inhibitors: PPARg; Znf202; Zfp125	[24–28]	Activators: ChREBP; HNF4α; PPARγ; FoxO1 Inhibitors: FXR; PPARα	[159]
Main sources	Liver, macrophages	[19, 24]	Liver, small intestine	[162]
Structure	57 aa-1 or 2 alpha helices	[36, 37]	79 aa-6 alpha helices	[163]
Glycosylations	None	[35, 36]	One: Thr74	[35]
Plasma distribution	TRL, HDL	[4]	TRL, IDL, HDL, (LDL)	[162]
Effect on TRL metabolism	Inhibition of LPL Inhibition of remnant clearance Increased VLDL assembly and secretion	[65, 67, 72]	Inhibition of LPL Inhibition of remnant clearance Increased VLDL assembly and secretion	[73, 164, 165]
Effect on HDL metabolism	Inhibition of CETP Activation of LCAT Increased PON-1 activity	[95, 100] [5, 6] [46]	Generation of pro-apoptotic HDL Inhibition of LCAT Inhibition of cholesterol efflux Impairment of anti-oxidant effect	[76, 166–168]
Effect on inflammation	Binding of LPS Increased macrophage activation	[139, 142]	Production of ROS, IL-6 and MCP-1 by endothelial cells NLRP3 activation in monocytes	[169, 170]
Effect on vascular wall	Apoptosis of VSMCs	[129]	Apoptosis of endothelial cells Adhesion of monocytes Proliferation of VSMCs Sub-endothelial retention of LDL	[169, 171–173]
Effect on diabetes	Reduced fat storage Improved insulin sensitivity	[108]	Apoptosis of beta-cells Induction of insulin resistance	[109, 110]

ApoC1 and apoC3 are small apolipoproteins originating from two different gene clusters. ApoC1 and apoC3 share some similarities, especially regarding their distribution among lipoprotein and their modulatory effect on TRL metabolism. However, their impact on HDL metabolism is opposite, with apoC1 increasing HDL levels and functionality and apoC3 promoting to the generation of dysfunctional HDL. Both proteins can affect inflammatory status and vascular biology but through different mechanisms, and with more numerous effects for apoC3. Finally, apoC1 and apoC3 seem to exert opposite effects on mechanisms linked with the onset of diabetes

*LXR* liver X receptor, *PPAR* peroxisome proliferator activated receptor, *ChREBP* carbohydrate-responsive element-binding protein, *FoxO1* forkhead box O1, *FXR* farnesoid X receptor, *TRL* triglyceride-rich lipoprotein, *IDL* termediate density lipoprotein, *LDL* low density lipoprotein, *HDL* high density lipoprotein, *LPL* lipoprotein lipase, *CETP* cholesteryl ester transfer protein, *LCAT* lecithin:cholesterol acyltransferase, *CD14* cluster differentiation 14, *TLR4* toll-like receptor 4, *ROS* reactive oxygen species, *IL-6* interleukin 6, *MCP-1* macrophage chemoattractant protein 1, *NLRP3* NOD-like receptor family, pyrin domain containing 3, *VSMCs* vascular smooth muscle cells

## Role of ApoC1 in HDL metabolism

In vitro studies have shown that apoC1 could play a role in HDL metabolism by modulating several plasma proteins and cellular receptors involved in the remodelling of these lipoproteins. Indeed, apoC1 partially activates lecithin-cholesterol acyltransferase (LCAT) [5, 6] which is involved in the maturation of HDL into spherical particles rich in esterified [74, 75]. This effect of apoC1 is opposite to that of apoC3 which inhibits LCAT activity (Table 1) [76]. Conversely, apoC1 is able to inhibit phospholipase A2 in vitro [7] which is responsible for increased HDL catabolism. However, none of these observations could be confirmed in vivo.

ApoC1 inhibits also hepatic lipase [77] which mediates triglyceride hydrolysis of cholesterol-rich lipoproteins, including HDL. Conde-Knape et al. [8] confirmed the modulating effect of apoC1 on hepatic lipase in vitro but only when apoC1 was located on HDL, and not on the surface of VLDL. In addition, as apoC2 and apoC3 were shown to inhibit hepatic uptake of cholesterol esters of HDL by the scavenger receptor B1 (SR-B1) [78], the effect of apoC1 on SR-B1 was also studied in vitro and in vivo in mice expressing high levels of apoC1 or in mice lacking apoC1. It was shown that apoC1 substantially diminished the uptake of HDL-bound cholesterol esters via SR-B1 [9].

Overall, these various effects of apoC1 on HDL metabolism (Fig. 3) suggest that the presence of apoC1 would lead to an increase in HDL-cholesterol levels, possibly by reduction of HDL catabolism. This could be confirmed in genetically engineered mice, with significantly lower HDL-C in apoC1 knock-out mice [9, 11] and increased HDL-C in mice overexpressing apoC1 [9, 10]. Once again, these effects should be confirmed in humans but clinical studies are lacking in this respect. Furthermore, studying the impact of apoC1 on HDL metabolism in mice suffers a major drawback since this species, unlike humans, is devoid of CETP which plays a major role in HDL metabolism by promoting the exchange of neutral lipids between HDL and triglyceride-rich lipoproteins. Importantly, apoC1 plays a major role in CETP activity, as discussed below.

## ApoC1, a physiological regulator of CETP

CETP is a lipoprotein-associated glycoprotein that enables the exchange of cholesterol esters (CE) and TG between HDL and TRLs [79, 80]. In vitro studies [81], observations in CETP transgenic mouse models [82] and clinical studies with genetic variants of CETP [83, 84] or treated with CETP inhibitors [85] consistently demonstrated that CETP promotes the net cholesterol enrichment of pro-atherogenic apoB-containing lipoproteins (VLDL and LDL) in parallel with a decrease in the cholesterol content of atheroprotective HDL. This pejorative effect of CETP on plasma lipoprotein profile raised the hypothesis that CETP might represent a relevant target to prevent cardiovascular diseases [86]. However, the pharmacological inhibitors of CETP that were tested so far in large clinical trials led to modest cardiovascular protection despite dramatic reductions in CETP activity [87].

Beside pharmacological compounds, the presence of an endogenous inhibitor of CETP in plasma has long been suspected. In vitro studies showed that CETP has a greater affinity for HDL than for LDL [88] and it could be demonstrated that, while LDL dose-dependently increases CETP activity, HDL exert an inhibitory effect at high doses [89]. These data suggest that a CETP inhibitory factor is associated with HDL. Although some studies found in vitro that apoA1 and apoA2 played a role in this inhibitory effect of HDL, others found a neutral or even an activating effect of apoAs on cholesteryl esters [90–96]. In vitro experiments comparing the modulatory effect of different purified apolipoproteins showed that apoC1 had the strongest inhibitory effect on CETP activity [97–99]. In comparison, apoC3 does not inhibit CETP in vitro [97]. These observations were confirmed in an exhaustive screening of HDL apolipoproteins allowed us to demonstrate that apoC1 was the main contributor to the inhibition of CETP when present as a component of HDL [95]. Furthermore, isolated apoC1 was found to decrease CETP activity in a concentration-dependant manner [95] while the effect of other apolipoproteins on CETP varied depending on the dose used [92]. The mechanisms involved in the inhibitory effect of apoC1 on CETP were investigated in vitro. We could show that the presence of apoC1 decreases the electronegativity of HDL, leading to a dissociation of HDL-CETP complexes [100]. This observation confirms the key role of surface potential in the interaction of CETP with lipoproteins [101, 102]. More into details, our group could demonstrate that this alteration of negative surface charge of HDL could be attributed to the presence of a positively charged, lysine-rich cluster in the C-terminal α helix of human apoC1, and blocking these amino-acid residues through chemically induced acetylation was associated with a significant decrease in the inhibitory effect of apoC1 on CETP [100]. Interestingly, rabbit apoC1, which unlike human or mouse apoC1 does not modify the electrostatic charge of HDL, is unable to inhibit CETP [12]. These studies thus showed that the electrostatic charge of apoC1 plays a major role in its ability to inhibit CETP.

The physiological relevance of CETP inhibition by apoC1 was first confirmed in vivo in transgenic mice expressing human CETP (CETP-Tg) crossed either with apoC1-knocked out mice (apoC1-KO mice), or with mice overexpressing human apoC1 (apoC1-Tg mice). In CETP-Tg/apoC1-KO mice, we observed that specific CETP activity was dramatically increased when compared with CETP-Tg mice. This was accompanied with significant decreases in the CE content and in the CE-to-TG ratio of HDL, in line with enhanced CETP-mediated neutral lipid exchanges between HDL and apoB-containing lipoproteins in the absence of apoC1 [103]. Ex vivo, the HDL of CETP-Tg/apoC1-KO mice interacted more readily with purified CETP than did HDL of CETP-Tg mice, even though these HDL only differed by their apoC1 content [103]. On the opposite, CETP-Tg-apoC1-Tg mice presented a twofold decrease in specific CETP activity compared with CETP-Tg mice [10]. More recently, we developed transgenic rabbits expressing human apoC1 which also helped to demonstrate that the presence of human apoC1 at physiological levels could rescue the lack of inhibitory potential of rabbit apoC1 against CETP, leading once again to an increase in plasma HDL-cholesterol [49].

The inhibitory effect of apoC1 on CETP activity was also confirmed in humans. Indeed, we observed significant negative correlations between plasma levels of apoC1 and CETP specific activity in three cohorts of normolipidemic subjects [12–14]. Although the correlation was less marked in patients with coronary artery disease (CAD) and treated with statins, apoC1 concentration was also positively correlated with the HDL to LDL-cholesterol ratio, suggesting once again that apoC1 might have a positive impact on plasma cholesterol profile through CETP inhibition [13]. However, this negative correlation between apoC1 levels and CETP activity was lost in CAD patients with hypertriglyceridemia or combined hyperlipidemia [13].

The presence of high amounts of TRLs might explain the loss of inhibitory potential of apoC1 in these dyslipidemic patients. Firstly, the abundance of triglyceride-rich lipoproteins, which are preferential acceptors of cholesterol esters from HDL [104, 105], might further stimulate the CETP-mediated cholesterol ester transport and thus overcome the inhibitory effect of apoC1 on CETP. Secondly, as mentioned above (see “Plasma levels and distribution of apoC1 between lipoproteins” Section), apoC1 is a highly exchangeable protein [4, 40] and the accumulation of VLDL in the bloodstream may lead to its preferential association with this lipoprotein class at the expense of HDL [3]. Importantly, we could show that apoC1 does not inhibit CETP when bound to VLDL [70]. Therefore, the decrease in the relative amounts of functional apoC1 present in the HDL might also explain the loss of negative correlation between apoC1 and CETP activity in subjects with high triglyceride levels. The fact that plasma triglyceride levels correlated positively with apoC1 bound to VLDL (i.e. apoC1 that cannot inhibit CETP), in a sub-group of healthy subjects reinforces the idea that the inhibitory of apoC1 is impaired when TRL accumulate [13].

## ApoC1 in diabetes

Plasma apoC1 levels are higher in type 1 and type 2 diabetic patients than in control subjects and we observed a positive correlation with plasma TG [14, 106, 107]. In the context of diabetes, apoC1 would thus appear as positively associated with some cardiometabolic risk factors. However, the causal relationship between high apoC1 levels and diabetes is questionable. In vivo studies in transgenic mice suggest that apoC1 overexpression protects against insulin resistance through reduced fat storage despite elevated plasma TG [108]. On the opposite, apoC3 appears as pro-diabetic by triggering pancreatic beta-cell apoptosis and by promoting insulin resistance via Toll-Like Receptor 2 (TLR2) activation in transgenic mouse models (Table 1) [109, 110]. In men with metabolic syndrome, elevated apoC1 levels were also associated with higher TG levels but lower visceral fat mass [111]. In a study conducted in a small cohort of subjects of American Indian or Mexican ancestry, a structural polymorphism of apoC1, the T45S variant, which is associated with a higher propensity of the protein to undergo N-terminal truncation, was found to be associated with higher prevalence of diabetes secondarily to the onset of obesity [112]. In a 5-year prospective study, although plasma levels of apoC1 correlated positively with a high risk of T2D, this link was lost after adjusting for TG levels [113]. The only genetic study which investigated a most common apoC1 gene polymorphism (rs11568822) in relation with diabetes failed to find a correlation [114]. These observations suggest that apoC1 itself is protective or neutral regarding diabetes risk, and that high apoC1 levels in diabetic subjects might rather be in relation with altered TG metabolism, i.e. increased production and/or lower catabolism of TRLs. Although apoC1, through is involvement in TRL clearance, might influence fat content in different tissues [11, 115], we observed an association between apoC1 plasma levels neither with adipose tissue areas or distribution (visceral or subcutaneous) nor with steatosis in patients with type 1 or type 2 diabetes, as the correlation between apoC1 and liver fat content disappeared after adjustment for triglyceride-level [106, 107]. High apoC1 levels in diabetic patients thus appear to be a consequence rather than a cause of impaired TG metabolism and fat storage in diabetic patients, but the precise mechanisms involved remain poorly understood. The elevation of plasma TG levels in most diabetic patients might explain only in part this phenomenon since our study in type 1 diabetics showed significantly elevated apoC1 despite TG levels similar to that of control subjects [107].

A loss of the negative correlation between apoC1 and CETP activity was observed in patients with type 1 or type 2 diabetes [14], similarly to what was reported CAD patients with hyperlipidemia. More strikingly, we also reported that CETP activity in both type 1 and type 2 diabetics was higher than that in controls (in agreement with the literature [116–120] despite higher apoC1 levels [14]. This reinforces the notion that apoC1 is dysfunctional in this population. Furthermore, CETP activity was significantly higher in type 2 diabetic patients than in type 1 diabetic patients [14]. This difference can be explained in part by TG levels, which directly stimulate CETP activity [104, 105] and which were 13% and 170% higher in type 1 and type 2 diabetic patients, respectively, than in controls. However, in a subgroup of type 1 diabetics with normal TG levels (below 1.7 mmol/L) CETP activity was still significantly higher than that in controls although TG levels in these 2 groups were not statistically different. In addition, in these type 1 diabetic patients with normal TG levels, there was no correlation between CETP activity and triglyceride levels. Therefore, the greater ability of CETP to transfer cholesterol esters in type 1 diabetic patients is probably related to mechanisms other than hypertriglyceridemia. Hyperglycaemia, which correlated with CETP activity in type 1 diabetic patients, arise as a possible explanation. It is proposed that glycation phenomena plays a key role in the loss of the inhibitory potential of apoC1 in diabetic patients. The presence of apoC1 with glycated lysine residues was reported in hyperglycemic diabetic subjects [121]. In vitro glycation of apoC1 was shown to modify its electrostatic properties, leading to a decrease in its ability to inhibit CETP activity [14]. Accordingly, we could confirm in vivo that the electrostatic properties of apoC1 were modified in type 1 diabetic patients [14]. These results are in agreement with the in vitro studies in which acetylation of lysine residues of apoC1 was accompanied by a reduction in its electropositive charge and by a weakening of its ability to inhibit CETP-induced cholesterol ester transport [100].

A meta-analysis reported an association between a polymorphism of apoC1 (rs4420638) and the risk of developing diabetic nephropathy [122]. In mice overexpressing apoC1, albuminuria and glomerulosclerosis with increased numbers of glomerular M1 macrophages were observed while no renal abnormalities were reported in wild-type mice [123]. In the clinical part of the same study, apoC1 was detected only in the kidneys from autopsied diabetic subjects and not in control subjects and the amount of apoC1 in glomeruli was significantly higher in patients with diabetic nephropathy than in those without diabetic nephropathy [123]. The authors suggest that apoC1 might play a local role in the development of diabetic nephropathy, probably by increasing macrophage activation in renal tissue.

Overall, diabetic states not only lead to elevated apoC1 levels, but also to the generation of structurally modified and dysfunctional apoC1. Beyond the use of apoC1 level as a new marker of diabetes severity, the restoration of apoC1 functionality might help to counteract the deleterious effects of increased CETP activity on plasma lipoprotein profile in this population at high cardiovascular risk.

## ApoC1 and atherosclerosis

**Fig. 4:**
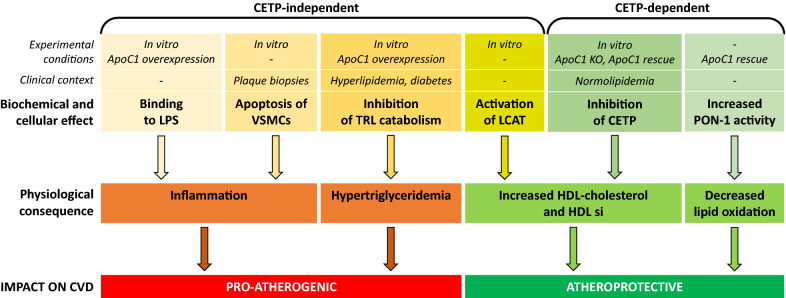
Pro-atherogenic and atheroprotective effects of apoC1.The biochemical and cellular effects of apoC1 were classified as dependent or not on the presence of CETP. The experimental conditions (in vitro experiments, animal models) or the clinical context (for studies in human subjects) in which the effects were observed are shown on the first two rows. *LPS* lipopolysaccharide, *VCSMs* vascular smooth muscle cells, *TRL*, triglycerides rich lipoproteins, *LCAT* lecithin cholesterol acyl transferase, *PON-1* paraoxonase-1, *CETP* cholesteryl ester transfer protein, *CVD* cardiovascular disease

Atherosclerosis is a complex, multifactorial disease which involves disturbed lipoprotein metabolism, inflammation, and alterations in the function of numerous cell types present in the vascular wall including endothelial cells, smooth muscle cells (VSMCs) or myeloid cells such as macrophages. ApoC1 is involved not only in many aspects of lipoprotein metabolism, but also in the regulation of inflammatory response and in the biology of some cell components of vascular wall (Fig. 4), making its net impact on atherosclerosis difficult to anticipate at first glance.

Several observations indicate that apoC1 is expressed by different cell types involved in the development of atherosclerotic plaque. The expression of the apoC1 gene was compared in the atheroma lesions of 10 subjects who underwent carotid or femoral endarterectomy and in healthy arteries harvested post mortem [124]. mRNA levels of apoC1 were higher in the carotid and femoral atherosclerotic lesions than in healthy arteries. Macrophages are involved in the initiation and the development of atherosclerotic lesions in vivo [125]. ApoC1 is highly expressed in macrophages, where its expression is tightly regulated by LXR which is deeply involved in the maintenance of cellular lipid homeostasis [24]. It was shown in vitro that human apoC1 increased cholesterol efflux from macrophages thanks to its α-helix conformation [126, 127], and could prevent cholesterol accumulation in macrophages exposed to modified LDL [128]. However, when bone marrow transplants from apoC1-KO or wild-type mice to apoE-KO mice were conducted to assess the specific effect of macrophage-derived apoC1 on the development of atherosclerosis, no difference was found for plasma either lipid levels or atherosclerotic lesions [128]. This indicates that apoC1 in macrophages is not likely to play a major role in the development of atherosclerotic plaques in vivo, despite its promoting effect on cholesterol efflux demonstrated in vitro. Beside macrophages, some studies reported a role of apoC1 in VSMC biology. It could be shown that apoC1 can induce apoptosis in cultured VSMCs via a mechanism involving neutral sphingomyelinase-ceramide pathway [129], and that apoC1 co-localized with markers of apoptosis (ceramide, caspase-3) in neo-intimal dissections in rabbit models of atherosclerosis [130]. These results suggest that at the local level, apoC1, like apoC3 (Table 1), could be involved in complications of the atherosclerotic process.

Inflammation plays a prominent role from initiation processes to the development of complications of atherosclerosis, as illustrated by the success of anti-inflammatory therapies to reduce major adverse cardiovascular events in high-risk patients [131, 132]. Inflammatory stimuli involved in atherosclerosis are very diverse and include, among others, oxidized lipoproteins and modified lipids but also damage- and pathogen- associated molecular patterns (DAMPS and PAMPS, respectively) [133]. The pro-apoptotic effect of apoC1 on VSMCs mentioned above can contribute to the generation of DAMPs from dying cells. Next to DAMPs, bacterial lipopolysaccharides (LPS, endotoxins) are major PAMPs triggering innate immune response. The presence of bacterial LPS in the circulation has long been known to aggravate atherosclerosis [134, 135]. In addition, LPS could also be found in atherosclerotic plaques from endarterectomy samples collected from patients with high cardiovascular risk, but not in healthy arterial wall [136–138]. ApoC1 was shown to interact with circulating LPS. Indeed, apoC1 can directly bind LPS via its α-helices and facilitates LPS presentation to innate immune receptors at the surface of macrophages, thereby enhancing inflammatory response [139]. Although this effect might be protective against infection and sepsis [140, 141], the presence of apoC1 was shown to enhance atherosclerotic lesion size and inflammatory status of the plaque in a genetically engineered mice where atherosclerosis was induced by repeated LPS injections [142]. ApoC3 was also reported to exert pro-inflammatory effects on components of the vascular wall, but via distinct mechanisms (Table 1).

The impact of apoC1 on atherosclerosis via its effect on plasma lipid metabolism was investigated in mice and rabbits. In two years old apoE-deficient mice expressing or not endogenous apoC1 [128], apoC1 expression was accompanied by a 70% increase in triglycerides levels and a 30% increase in cholesterol levels which were essentially related to an increase in triglycerides and cholesterol in the VLDL. Atherosclerotic lesions in mice expressing apoC1 were 87% greater than in the other mice. The macrophage content of the atherosclerotic lesions was not different between the 2 populations of mice. Data from this study suggest that systemic apoC1 aggravates atherosclerosis, probably via the accumulation of TRLs, since apoC1 specifically derived from macrophages had no effect on the pathology [128]. However, studying the lipid mediated effects of apoC1 on atherosclerosis in mice is hampered by the absence of CETP in this species, which leads to overlooking the possible impact of apoC1 on CETP-mediated remodelling of HDL as occurs in humans. ApoC1 and its effect on atherosclerosis were studied in rabbits by our group more recently. Rabbits indeed appear to be a better model than mice for the study of atherosclerotic effects of apoC1, because (1) rabbits fed with cholesterol-rich diet spontaneously develop atherosclerotic lesions, as humans but not as mice [143] (2) plasma lipid profile of rabbits is closer to humans than the one of mice and (3) rabbits, like humans, express endogenous, active CETP [144]. In this study, wild type rabbits were compared with transgenic rabbits expressing human apoC1 (HuApoC1-Tg rabbits), in terms of atherosclerosis, plasma lipid and lipoprotein profiles [49]. After 8 weeks of cholesterol-rich diet, all rabbits experienced a massive increase of plasma cholesterol levels in apoB-containing lipoproteins, without any significant difference in hypercholesterolemia between the two groups of rabbits. Of note, plasma TG levels did not differ either between both groups while HDL-cholesterol was higher in HuApoC1-Tg rabbits than in wild-type animals. This increase was associated with an impairment of HDL to serve as substrates for CETP in HuApoC1 rabbits. Human apoC1 prevented extension of aortic atherosclerotic lesions in rabbits fed with cholesterol-rich diet while, on the opposite, CETP activity was found to be correlated positively with the extent of atherosclerosis. This protective effect of human apoC1 was not due to decreased inflammatory status or increased cholesterol efflux from macrophages but was associated with a decrease in markers of lipid oxidation in plasma. This study suggests that, when CETP is expressed as in humans, moderate elevation of plasma apoC1 level leads to increased levels of atheroprotective HDL. This atheroprotective mechanism is a direct consequence of CETP inhibition and is mostly operative in the absence of concomitant, potentially deleterious changes in TRL metabolism. From this respect, apoC1 drastically differs from apoC3 which, in addition to its hypertriglyceridemic effect, has a pejorative impact on HDL functions such as cholesterol efflux and protection against apoptosis or oxidation (Table 1).

In humans, the net impact of apoC1 on atherosclerosis was assessed in several genetic studies. The most common polymorphism at the apoC1 gene (HpaI, rs11568822) consists in a 4 base pair insertion/deletion located in the proximal promoter and resulting in a high and low gene expression in vitro, respectively [145]. The insertion variant was found to be associated with metabolically unhealthy phenotypes in obese people or in people with normal weight [146] as well as with an elevation of triglyceride levels [114, 147] thereby consolidating the possible impact of high apoC1 levels on TRL metabolism in humans. However, none of these studies could find an association between this polymorphism and coronary artery disease or myocardial infarction. In a prospective study in more than 2767 subjects, the analysis of additional polymorphisms at the apoC1 gene evidenced a slight effect on coronary heart disease [15]. Additional polymorphisms located in the ApoE-apoC1 locus were also identified as possibly linked with altered plasma lipid levels or atherogenic risk by different bioinformatics approaches including genome wide association studies (GWAS) or protein network analysis [16, 148, 149]. However, these studies underlie the fact that these apoC1 polymorphisms are in strong linkage disequilibrium with apoE variants which could explain the majority of cardiovascular disease (CVD) risk [18], thus questioning the real impact of apoC1 polymorphisms per se on atherosclerosis [15–17].

The possible association between circulating apoC1 and cardiovascular risk was also explored in humans. A first study was conducted in patients with metabolic syndrome and demonstrated that high plasma levels of apoC1 were associated with increased carotid wall thickness only in the subgroup presenting with systemic inflammation (hsCRP > 3 mg/L) [150]. A second group set up a prospective study in patients with heart failure and measured plasma apoC1 using LC-MRM-MS at inclusion before a 3 year follow-up. Higher levels of plasma apoC1 were found in patients who died from cardiovascular diseases when compared to those who did not. This difference was observed whereas triglycerides, total cholesterol, HDL cholesterol and LDL cholesterol levels after three years of follow-up were similar between the two groups [47]. These pejorative correlations between plasma apoC1 and atherosclerosis or cardiovascular death were observed in contexts that are critical for atherosclerosis and cardiovascular events, i.e. metabolic syndrome and inflammation on the one hand and heart failure on the other hand. Interestingly, the presence of structurally altered forms of apoC1 was reported after MALDI-TOF analyses on serum from patients with CAD. One study showed indeed that CAD patient displayed a truncated form of apoC1 which appeared to be more susceptible for oxidative modifications [151]. Accordingly, another group also observed the presence of oxidized apoC1 in CAD patients but not in healthy control subjects [152]. Whether these structural modifications contribute to, or are a consequence of atherosclerosis is not known but, in a similar manner to what was observed with glycation phenomena in diabetic subjects [14], they might be associated with alterations of apoC1 functionality. In line with this latter hypothesis, it could be shown that the negative correlation between apoC1 and CETP activity was less marked in CAD patients than in healthy subjects [13].

Postprandial elevation of triglycerides is frequently observed in normolipidemic coronary heart disease patients. Some postprandial disturbances of lipid metabolism may thus serve as early markers of cardiovascular risk. It was shown that the lipid and apolipoprotein composition of VLDL was modified in the postprandial period. In normolipidemic male subjects with CAD, it was shown that the apoC1 content of VLDL was 50 to 100% higher than that in control subjects in the postprandial period [52]. The number and the composition of VLDL and of chylomicron remnants in the postprandial period were further studied in thirty 50-year-old normolipidemic men with no cardiovascular history [153]. The subjects with early signs of carotid atherosclerosis (increased intima-media thickness, IMT) did not show greater postprandial response, but they presented twofold increased levels of apoC1 in the chylomicron remnants and in VLDL when compared with the subjects with normal intima-media thickness [153]. Furthermore, the apoC1 content of postprandial TRL was independently correlated with IMT in a population of middle aged men which had all an apoE3/E3 genotype [154]. However, other studies evidenced a higher enrichment of apoC1 in VLDL in patients with carotid atherosclerosis compared to controls [155] and a positive correlation between apoC1 enrichment of VLDL and carotid plaque area [71] for VLDL analysed in the fasting state, but not in the postprandial phase. Although the relative contribution of remnants of the postprandial phase and fasting VLDL is still unclear, these data indicate that apoC1 content of TRLs could be an early marker of cardiovascular risk. These observations further support tight connection between apoC1 and TRLs although the causative relationship between apoC1 enrichment of TRL and elevated TG levels still needs to be explored. Abnormalities of apoC1 distribution during hypertriglyceridemic states, with preferential association with TRLs [71], could once again be linked with alterations of apoC1 functionality, redirecting apoC1 function towards inhibition of TG catabolism, thereby increasing CVD risk [156] at the expense of its inhibitory effect against CETP when present in the HDL [157]. Interestingly, it was shown in a prospective family-based study that plasma levels of apoC1 were positively associated with large HDL at birth and in infants, while it was correlated with TG levels, but not HDL, in adults [158]. It suggests that a progressive shift of apoC1 location and function occurs along life with an increase of the relative importance of its inhibitory effect on TG metabolism along the build-up of cardiometabolic risk.

## Conclusion

Data available for apoC1 principally concern in vitro studies and transgenic animal models. Strikingly, it appears that the balance between protective and deleterious effects of apoC1 mostly rely on the presence of CETP in the experimental models (Fig. 4), as illustrated, among others by opposite effects human apoC1 on atherosclerosis between mice [64] and rabbits [49]. Beyond animals, the very limited amount of studies conducted in humans might also explain why the precise role of apoC1 on lipid metabolism and cardiovascular risk remains unclear. On the one side, several data indicate that apoC1 seems to be pro-atherogenic by increasing TG level which may be explained by the inhibition of binding to LDL-R, to LRP and to VLDL-R, the inhibition of LPL or the stimulation of VLDL production. On the other side, apoC1 associated with HDL seems to be anti-atherogenic in HDL metabolism by inhibiting CETP, when VLDL-bound apoC1 is unable to inhibit CETP. In addition, apoC1 loses its ability to inhibit CETP in a context of hyperlipidemia and diabetes.

We can hypothesize that apoC1 may have different functions according to its distribution between lipoproteins (with a pro-atherogenic effect when associated with VLDL and an anti-atherogenic effect when associated with HDL) and that qualitative changes of apoC1 occurring in clinical situations as diabetes and atherosclerosis may also modify its properties and expression pattern. In this aspect, apoC1 differs from another small, highly exchangeable apolipoprotein, i.e. apoC3. Indeed, although both proteins impair plasma TG clearance when bound to TRLs, their impact on HDL functionality is opposite: apoC3 exerts a negative effect and apoC1 a positive effect on the atheroprotective functions of HDL, including its inhibitory effect on CETP (see Table 1). Overall, while apoC1 harbors either beneficial or deleterious effects on lipoprotein metabolism and cardiometabolic diseases, apoC3 appears in most cases as a risk factor. Accordingly, the strong positive correlations between apoC3 levels or genetic variants with cardiovascular risk [159] led to the development of multiple anti-apoC3 therapeutic strategies [160]. In contrast, using apoC1 as a therapeutic target might be more complex.

Despite the possible beneficial use of apoC1 as a CETP inhibitor against atherosclerosis, strategies aiming at inducing very high plasma levels of apoC1 would not be relevant because of the possible hypertriglyceridemic effect [10]. Importantly however, it must be borne in mind that beneficial effects of apoC1 on atherosclerosis might be reached on the long term even when keeping its plasma levels within a physiological range as suggested by our studies in transgenic rabbits [46]. Alternatively, it is tempting to speculate that therapeutic interventions that could prevent the sequestration of apoC1 in TRLs or its structural modifications (truncation, oxidation, glycation) could improve its inhibitory function against CETP and restore, maintain or magnify its anti-atherogenic function.

Other studies are still necessary, particularly in humans, to determine the precise role of apoC1 lipoprotein metabolism but also for its possible pleiotropic effects on inflammation and vascular wall biology, in order to get a comprehensive view of its potential impact in the development of atherosclerosis.

## Data Availability

Not applicable.

## Abbreviations

ApoC1: Apolipoprotein C1
CAD: Coronary artery disease
CE: Cholesteryl ester
CETP: Cholesterol Ester Transfer Protein
CVD: Cardiovascular disease
DAMPs: Damage- associated molecular patterns
HDL: High density lipoprotein
HCR-1/HCR-2: Hepatic control regions-1 and -2
IMT: Increased intima-media thickness
LCAT: Lecithin-cholesterol acyltransferase
LDL: Low density lipoprotein
LDL-R: Low density lipoprotein receptor
LRP: LDL receptor related protein
LPL: Lipoprotein lipase
LPS: Lipopolysaccharide
LXR: Liver X receptor
ME-1 and ME-2: Multienhancers 1 and 2
PAMPs: Pathogen-associated molecular patterns
SR-B1: Scavenger receptor B1
TG: Triglycerides
TRL: Triglyceride-rich lipoprotein
VLDL: Very low density lipoprotein
VSMCs: Vascular smooth muscle cells
